# The Role of Vitamin E in Immunity

**DOI:** 10.3390/nu10111614

**Published:** 2018-11-01

**Authors:** Ga Young Lee, Sung Nim Han

**Affiliations:** 1 Department of Food and Nutrition, College of Human Ecology, Seoul National University, Seoul 08826, Korea; lgykiki90@snu.ac.kr; 2 Research Institute of Human Ecology, Seoul National University, Seoul 08826, Korea

**Keywords:** vitamin E, macrophages, T cells, dendritic cells, immunomodulation, infection

## Abstract

Vitamin E is a fat-soluble antioxidant that can protect the polyunsaturated fatty acids (PUFAs) in the membrane from oxidation, regulate the production of reactive oxygen species (ROS) and reactive nitrogen species (RNS), and modulate signal transduction. Immunomodulatory effects of vitamin E have been observed in animal and human models under normal and disease conditions. With advances in understating of the development, function, and regulation of dendritic cells (DCs), macrophages, natural killer (NK) cells, T cells, and B cells, recent studies have focused on vitamin E’s effects on specific immune cells. This review will summarize the immunological changes observed with vitamin E intervention in animals and humans, and then describe the cell-specific effects of vitamin E in order to understand the mechanisms of immunomodulation and implications of vitamin E for immunological diseases.

## 1. Vitamin E: Definition, Structure, Sources, and Functions

### 1.1. Definition and Structure

**Figure 1:**
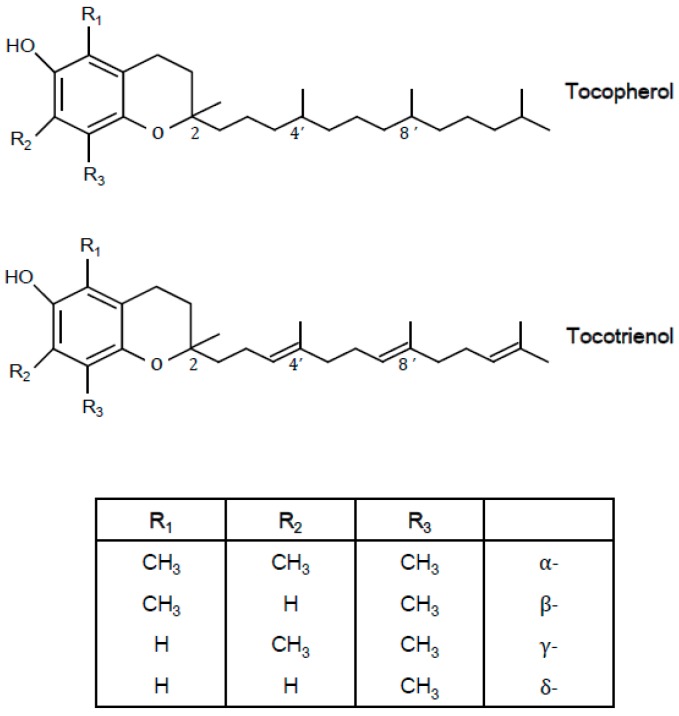
The structures of tocopherol and tocotrienols.

Vitamin E is the collective term for four tocopherols (α-, β-, γ-, and δ-tocopherols) and four tocotrienols (α-, β-, γ-, and δ-tocotrienols) found in food. These forms have antioxidant activities, but cannot be interconverted, and only α-tocopherol meets the human vitamin E requirement [1]. Tocopherols have a chromanol ring and a phytyl tail, while tocotrienols have a chromanol ring and an unsaturated tail. The α-, β-, γ-, and δ- forms differ in the number and position of methyl groups on the chromanol structure. Natural tocopherols have only *RRR* stereochemistry, but synthetic tocopherols are mixtures of eight stereoisomers (*RRR-, RSR-, RRS-, RSS-, SRR-, SSR-, SRS-, SSS-*), because there are three asymmetric carbon atoms (2*R*, 4’*R*, 8’*R*) present in the phytyl tail. The structures of tocopherols and tocotrienols are shown in Figure 1.

### 1.2. Sources

The major dietary sources of vitamin E are vegetable oils. Nuts are good sources of vitamin E as well [2]. Soybean, sunflower, corn, walnut, cottonseed, palm, and wheat germ oils contain relatively higher amounts (more than approximately 50 mg vitamin E/100 g oil) of vitamin E than other oils. The proportions of α-, β-, γ-, and δ-tocopherols vary depending on the oil type. Safflower and sunflower oils are high in α-tocopherol, soybean and corn oils contain mainly γ-tocopherol, and cottonseed oil contains similar proportions of α- and γ-tocopherols. Therefore, the types of oils consumed through the diet affect the dietary intake levels of α-tocopherol. Vitamin E supplements are quite popular and contribute considerably to vitamin E intake among some populations. Either natural or synthetic forms of α-tocopherol are used as supplements.

Despite the relatively higher intake of γ-tocopherol from the diet than α-tocopherol, α-tocopherol is the major form of vitamin E in the circulation because α-tocopherol transfer protein (α-TTP) has the preferential binding affinity for α-tocopherol. α-TTP is involved in the transfer of α-tocopherol to the plasma membrane [1].

### 1.3. Functions

Vitamin E is a major fat-soluble antioxidant that scavenges peroxyl radicals and terminates the oxidation of polyunsaturated fatty acids (PUFAs). In the presence of vitamin E, peroxyl radicals react with α-tocopherol instead of lipid hydroperoxide, the chain reaction of peroxyl radical production is stopped, and further oxidation of PUFAs in the membrane is prevented [1]. Tocopheroxyl radicals—produced from α-tocopherol and peroxyl radicals—are reduced by vitamin C or glutathione, form tocopherol dimers, undergo further oxidation, or act as prooxidants. The antioxidant activity of vitamin E may be responsible for the regulation of several enzymes involved in signal transduction because the activity of signaling enzymes is regulated by the redox state.

Vitamin E inhibits protein kinase C (PKC) activity by increasing PKC-α dephosphorylation through the activation of protein phosphatase 2A. The inhibition of PKC by vitamin E has been reported in various cells, and consequently, the inhibition of platelet aggregation; reduced proliferation of monocytes, macrophages, neutrophils, and vascular smooth muscle cells; and decreased superoxide production in neutrophils and macrophages have been observed [3,4].

Vitamin E may directly bind to the enzymes involved in the generation of lipid mediators or to the transport proteins involved in signal transduction. Vitamin E may affect the membrane protein interaction and translocation of the enzymes to the plasma membrane and therefore change the activity of signal transduction enzymes [4].

## 2. Modulation of Immune Responses and Infectious Diseases by Vitamin E Supplementation

### 2.1. Immune Responses in Animals

Dietary interventions of vitamin E at supplemental levels have been shown to enhance cell-mediated and humoral immune responses in various species of animals. Increased lymphocyte proliferation, immunoglobulin levels, antibody responses, natural killer (NK) cell activity, and interleukin (IL)-2 production have been reported with vitamin E supplementation (Table 1).

### 2.2. Immune Responses in Humans

In humans, many intervention studies have reported increased lymphocyte proliferation in response to mitogenic stimulation, enhanced delayed type hypersensitivity (DTH) response, increased IL-2 production, and decreased IL-6 production with vitamin E supplementation above the recommended levels. However, some studies showed no difference or decreased lymphocyte proliferation responses and decreased chemiluminescence. (Table 2). Differences in dose of vitamin E supplementation used, magnitude of vitamin E level changes with supplementation, age of subjects, and methodology (determination of antibody levels with or without specific vaccination) might have contributed to the different results observed.

### 2.3. Infectious Diseases in Animals

The immunostimulatory effect of vitamin E has resulted in enhanced resistance against several pathogens. Animal studies in which infectious disease models were used to test the effects of vitamin E supplementation are listed in Table 3.

The mechanisms involved with protection against infectious agents were increased macrophage activity and antibody (Ab) production for *D. pneumoniae* type 1 [5], and higher NK activity and Th1 response for influenza virus [6,7].

### 2.4. Infectious Diseases in Humans

In humans, the effects of vitamin E on the natural incidence of infectious diseases have been determined in several studies (Table 4). Many studies provided evidence that the immunostimulatory effects of vitamin E confer improved resistance to infections. However, the magnitudes of the effects were rather small, and in some studies, positive effects were only observed in subgroups of subjects.

## 3. Vitamin E and Immune Cells

**Figure 2:**
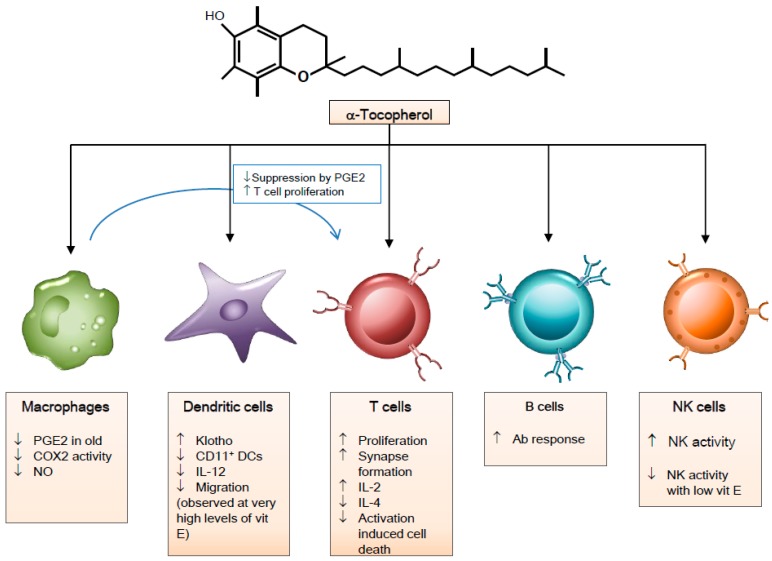
Immunomodulatory effects of vitamin E on immune cells. Abbreviations: PGE_2_, prostaglandin E_2_; COX2, Cyclooxygenase 2; NO, Nitric oxide; CD, Clusters of Differentiation; DCs, Dendritic cells; IL-12, Interleukin-12; Ab, antibody; NK, Natural killer.

The immunomodulatory mechanisms of α-tocopherol in immune cells are depicted in Figure 2.

### 3.1. Macrophages

Macrophages, important effector cells in the innate immune response, serve as antigen presenting cells (APC) and regulate NK cells and T cells by producing cytokines, reactive oxygen species (ROS), reactive nitrogen species (RNS), and prostaglandins. Cytokines produced by T cells and other immune cells can shift the macrophages into different populations with distinct physiologies [60].

The effects of vitamin E on prostaglandin (PG)E_2_ production by macrophages from the aged have been suggested as one of the mechanisms by which vitamin E improves the age-associated decrease in the T cell-mediated immune response [61]. In a co-culture experiment in which purified T cells and macrophages from young and old mice were cultured together, T cells from young mice showed suppressed proliferation and IL-2 secretion when cultured with macrophages from old mice. When macrophages from old mice were pre-incubated with 10 µg/mL vitamin E for 4 h, co-cultures of old macrophages and young T cells showed significant improvement in proliferation. Vitamin E pre-incubation of old macrophages improved proliferation and IL-2 production in co-cultures of old macrophages and old T cells [62]. Macrophages from old mice produced significantly higher levels of PGE_2_, which was due to higher cyclooxygenase (COX) activity. Macrophages from old mice expressed higher levels of inducible COX2 protein and mRNA [63]. These increases in PGE_2_ synthesis and COX activity were lowered by in vivo vitamin E supplementation [64]. Macrophages isolated from old mice fed a diet containing 500 ppm vitamin E for 30 days produced lower amounts of PGE_2_ and had lower COX activity than those from old mice fed a control diet containing 30 ppm vitamin E, but the COX2 mRNA levels and protein expression of the control and supplemented groups did not differ. Thus, vitamin E’s effect on COX activity seemed to be through post-translational mechanisms rather than through its effect at transcriptome or translational levels. In a subsequent study, it was shown that vitamin E reduced COX activity in macrophages from old mice by decreasing peroxynitrite production [65]. The inhibition of COX activity by vitamin E in old mice disappeared specifically with the addition of a nitric oxide (NO) donor in the presence of a superoxide to elevate peroxynitrite levels in the macrophage culture. There is a complex interplay between the nitric oxide synthase (NOS) and COX pathways and NO increases COX2 activity, which seems to be due to the NO preventing self-deactivation of COX by the superoxide as NO interacts with the superoxide [66].

In vivo supplementation of vitamin E (1500 IU d-α-tocopheryl acetate/day for 16 weeks) in allergic asthmatic patients prevented the suppression of alveolar macrophage nuclear factor (erythroid-derived 2)-like 2 (NRF2) activity after allergen challenge [67]. This study presented the possibility of vitamin E’s protective role in allergies and asthmas through regulation of macrophage NRF2 activity, but, further studies are needed to confirm the findings because of the small number of patients (nine mild non-smoking allergic asthmatics) and the lack of appropriate controls.

### 3.2. Natural Killer Cells

NK activity seems to be related with vitamin E status. The NK activity of a boy with Shwachman syndrome who had a severe vitamin E deficiency was low, but improved after eight weeks of 100 mg/d α-tocopherol supplementation. When α-tocopherol supplementation was stopped, NK activity and CD16^+^ CD56^+^ cells decreased. NK activity and CD16^+^ CD56^+^ cells were restored upon resuming eight weeks of 100 mg/d α-tocopherol supplementation [68]. In 37 women aged 90–106 years old, NK cell cytotoxicity was positively associated with plasma vitamin E concentration [69]. A two-week supplementation of 750 mg vitamin E in colorectal cancer patients resulted in increased NK activity in six out of seven patients. Vitamin E treatment did not result in changes in perforin expression or IFN-γ production; therefore, mechanisms of improved NK activity by vitamin E could not be determined from the study [70].

NO appears to be involved in the impairment of NK cell function. Co-culture of NK cells and myeloid-derived suppressor cells (MDSCs) showed that NK cell cytotoxicity and IFN-γ were impaired by MDSCs and that the inhibition of inducible nitric oxide synthase (iNOS) rescued the impairment by MDSCs. Exposure of NK cells to NO by treatment with an NO producer caused the nitration of tyrosine residues on CD16^+^ NK cells. These results suggested that MDSCs impair NK cell function via the production of NO and the nitration of protein tyrosine residues [71]. Vitamin E might exert its effects on NK cell function by modulating NO levels.

### 3.3. Dendritic Cells

Dendritic cells (DCs) are effective antigen-presenting cells that recognize pathogens and present pathogen-derived antigens to T cells. The interaction of DCs with pathogen-associated molecular patterns (PAMPs) or damage-associated molecular patterns (DAMPs) elicits the activation and maturation of DCs. The increased expression of surface major histocompatibility complex (MHC) molecules and co-stimulatory molecules and the increased production of cytokines occur with the activation of DCs, which allows the effective induction of the T cell response [72,73,74]. DCs are also involved in tolerance and autoimmunity. DCs might promote tolerance by the generation of Treg cells and/or by the induction of T cell unresponsiveness. DCs might be involved in the pathogenesis of autoimmune disease by promoting the priming or differentiation of self-reactive T cells [72]. Therefore, understanding the regulation of DCs by vitamin E will provide insight into the mechanisms of vitamin E’s immune response modulation and implications of vitamin E in immunological diseases.

Several studies have shown that vitamin E could regulate the maturation and functions of DCs. Tan et al. [75] investigated the effects of α-tocopherol and vitamin C, alone or in combination, on the phenotype and functions of human DCs generated from peripheral blood mononuclear cells (PBMCs). During the differentiation of human PBMCs into DCs, various concentrations of α-tocopherol were treated in culture starting from day 2, cells were stimulated on day 5, and then the surface phenotype was determined on day 6. The expression of human leukocyte antigen(HLA)-DR, CD40 CD80, and CD86 appeared to be increased with lower concentrations of α-tocopherol (<0.05 mM), but the combination of vitamin E and C prevented DC activation, as the upregulation of surface markers was not observed. DCs treated with 0.5 mM vitamin E and 10 mM vitamin C showed lower levels of intracellular ROS and inhibition of the nuclear factor (NF)-κB, PKC, and p38 mitogen-activated protein kinase (MAPK) pathways. When bone marrow-derived dendritic cells (BMDCs) from Balb/c mice were treated with 500 µM of α-tocopherol for 2 h, upregulation of phosphorylated inhibitor of κB (IκB) by lipopolysaccharide (LPS)-stimulation was suppressed. Vitamin E treatment for 24 h resulted in a reduced number of CD11^+^CD86^+^ cells and ROS-positive cells, lower production of IL-12p70 and TNF-α, and decreased transwell migration of BMDCs. These effects of vitamin E on BMDCs were partly dependent on Klotho expression. Vitamin E treatment on BMDCs resulted in higher Klotho transcript and protein levels, and silencing of Klotho by transfection of *Klotho* siRNA abolished the inhibitory effects of vitamin E on IL-12p70 production, number of ROS-positive cells, and DC migration [76]. Klotho is a membrane protein that has been shown to mediate calcium transport into the cells; regulate intracellular signaling pathways such as p53/p21, cyclin adenosine monophosphate (cAMP), PKC, and Wnt; and inhibit the NF-κB pathway [77]. Therefore, the upregulation of Klotho by vitamin E could be one of the mechanisms by which vitamin E modulates NF-κB mediated DC function and maturation. However, the level of α-tocopherol used for in vitro treatment (500 µM) was high and, therefore, further research is needed to elucidate the physiological relevance of vitamin E treatment on the expression of Klotho and its involvement in the modulation of DC function.

In vivo supplementation of α-tocopherol at 150, 250, and 500 mg/kg diet in allergic female mice reduced the lung CD11b^+^ DCs and mRNA levels of IL-4, IL-33, thymic stromal lymphopoietin (TSLP), eotaxin 1 (CCL11), and eotaxin 2 (CCL24) in allergen challenged pups. Furthermore, when BMDCs from 10-day-old neonates born to a control female were treated with 80 µM α-tocopherol for 24 h, the number of CD45^+^ CD11b^+^ CD11^+^ DCs and the number of CD45^+^ CD11b^+^ CD11c^+^ Ly6c^−^ MHCII^−^ DCs were reduced. Maternal supplementation with α-tocopherol was effective in decreasing allergic responses in offspring from allergic mothers by affecting the development of subsets of DCs that are critical for allergic responses [78]. On the other hand, γ-tocopherol supplementation exerted an opposite response in the same model. In vivo supplementation of γ-tocopherol at 250 mg/kg diet in allergic female mice resulted in a higher number of lung eosinophils, a higher number of lung CD11c^+^ CD11b^+^ DCs, and higher levels of lung lavage CCL11 in the offspring [79].

Modulation of the immune response by vitamin E has been observed in animal and human studies, and DCs play a critical role in bridging innate and adaptive immune systems and initiating adaptive immune responses. Despite the importance of DCs’ role in adaptive immune responses and in diseases such as autoimmune diseases, few studies have investigated the DC-specific effect of vitamin E.

### 3.4. T Cells

The effects of vitamin E on immune cells have been studied the most with T cells. The dysregulation of immune function occurs with aging and the most significant changes are observed in T cells. Age-associated changes in T cells include, but are not limited to, (1) defects in T cell receptor (TCR) signal transduction such as a decrease in linker for the activation of T cells (LAT) phosphorylation by zeta chain of T cell receptor associated protein kinase 70 (ZAP-70), (2) decreased intracellular influx of calcium following stimulation, (3) diminished synapse formation, (4) diminished activation of the mitogen activated protein kinase (MAP kinase) pathway, (5) decreased nuclear factor of activated T-cells (NFAT) binding activity, and (6) a shift of the T cell population toward memory T cells [80]. As a result, diminished production of IL-2 and reduced proliferative capacity of naive T cells are observed and impaired T cell functions contribute to increased susceptibility to infectious diseases and poor response to immunization.

Vitamin E has been shown to increase the cell division and IL-2 producing capacity of naïve T cells, increase the percentage of T cells capable of forming an effective immune synapse, and reverse the age-associated defect in the phosphorylation of LAT in T cells from old animals [81,82,83].

In vitro pre-incubation with 46 µM vitamin E for 4 h increased proliferation and IL-2 production in T cells purified from old mice stimulated with anti-CD3 and anti-CD28. Increased IL-2 production was due to both an increase in the number of activation-induced IL-2^+^ cells and an increase in the level of IL-2 accumulated per cell. Vitamin E specifically increased the naive T cells’ ability to progress through the cell division cycle in old mice [81]. The gene expression profile of T cells isolated from young and old mice fed a diet supplemented with 500 ppm vitamin E for four weeks provided evidence that vitamin E influences cell cycle-related molecules at the gene expression level. Higher expression of cell cycle-related genes *Ccnb2*, *Cdc2*, and *Cdc6* was observed in stimulated T cells from old mice fed the vitamin E-supplemented diet compared with those fed the control diet, which was not observed in young mice [84]. Cyclin B2, encoded by *Ccnb2*, binds to cyclin-dependent kinase 1 (also known as Cdc2) and regulates the events during both the G_2_/M transition and progression through mitosis. Cdc6 is a key regulator in the early steps of DNA replication, as the binding of Cdc6 to chromatin is a necessary and universal step in the acquisition of replication competences [85]. These alterations in the expression of cell cycle-related genes observed with vitamin E might contribute to vitamin E improving the proliferative ability of old T cells.

Marko et al. [82] showed that pre-incubation of CD4+ T cells isolated from old T cells with 46 µM vitamin E for 4 h increased the percentage of CD4^+^ T cells displaying effective immune synapses. Redistribution of Zap70, LAT, Vav, and phospholipase Cγ (PLCγ) into immune synapse increased significantly with vitamin E treatment. This change was confirmed with in vivo supplementation of vitamin E. In old mice fed a diet containing 500 ppm vitamin E for eight weeks, LAT and Vav showed significantly higher redistribution into the T cell/APC contact area when purified CD4^+^ T cells were stimulated with murine CD3ε hybridoma. In a subsequent study, it was shown that vitamin E could reverse the age-associated defect in the phosphorylation of LAT on tyrosine 191 [83]. The phosphorylation of LAT is required for the recruitment of adaptor and effector proteins. Therefore, it plays a pivotal role in the assembly of microcluster structures in the initiation of T cell activation signals. This evidence suggests that vitamin E can modulate the early stages of T cell activation.

Vitamin E seems to modulate Th1 and Th2 responses. The polarization of CD4 T cells to T helper (Th)1 or Th2 cells has implications for the protection against different pathogens (intracellular vs. extracellular pathogens) and the development of different types of chronic diseases (inflammatory vs. allergic diseases). PBMCs isolated from allergic donors treated with vitamin E (12.5–50 µM) showed dose-dependent decreases in IL-4 production [86]. IL-4 mRNA levels in activated PBMCs were downregulated by 25 µM vitamin E treatment. Jurkat T cells treated with 50 µM vitamin E exhibited downregulation of IL-4 promoter activity, which might be related to vitamin E blocking the interaction of transcription factors with PRE-1 and P1. In vivo supplementation of vitamin E enhancing the Th1 response has been observed in mice infected with influenza virus and in colorectal cancer patients [6,87]. In colorectal cancer patients, two weeks of supplementation with 750 mg vitamin E led to an increased frequency of IL-2 producing CD4+ T cells and increased IFN-γ production [87]. In old mice infected with influenza virus, 500 ppm vitamin E supplementation for eight weeks prior to infection lowered the viral titer in the lung, and this protective effect of vitamin E was associated with the enhancement of Th1 response. IFN-γ production levels correlated negatively with viral titer, and old mice fed a vitamin E-supplemented diet produced significantly higher levels of IFN-γ and IL-2 [6]. The gene expression profile of T cells isolated from young and old mice fed a diet supplemented with 500 ppm vitamin E for four weeks provided evidence that vitamin E influences the Th1/Th2 balance at the gene expression level. The increase in IL-4 expression following stimulation was lower in T cells from old mice fed the vitamin E-supplemented diet compared with those fed the control diet, and the ratio of IFN-γ and IL-4 expression levels was significantly higher in the vitamin E group than in the control group [84].

Vitamin E can affect activation-induced cell death in T cells. In vitro treatment of primary human T cells with 25 µM vitamin E suppressed CD95L expression and activation-induced cell death [88]. The reduction of CD95L mRNA levels and the proportion of CD95L-positive cells were related to the suppression of NF-κB and AP-1 binding to the CD95L promoter target site by vitamin E. On the other hand, α-tocopheryl succinate was shown to trigger apoptosis in Jurkat cells with caspase-activation involved [89].

### 3.5. B Cells

Vitamin E supplementation has been reported to enhance humoral responses. Higher antibody responses have been observed in animals and humans [19,27]. However, it is hard to differentiate whether vitamin E’s direct effect on B cells or indirect effect through T cells contributes to higher antibody responses.

## 4. Conclusions

Vitamin E has been shown to enhance immune responses in animal and human models and to confer protection against several infectious diseases. Suggested mechanisms involved with these changes are (1) the reduction of PGE_2_ production by the inhibition of COX2 activity mediated through decreasing NO production, (2) the improvement of effective immune synapse formation in naive T cells and the initiation of T cell activation signals, and (3) the modulation of Th1/Th2 balance. Higher NK activity and changes in dendritic function such as lower IL-12 production and migration were observed with vitamin E, but underlying mechanisms need to be further elucidated

Several considerations are warranted for the advancement in our understanding of vitamin E’s role in immunity. For in vitro studies to support implications for the regulation of immunological diseases, the physiological relevance of vitamin E levels used for treatment should be considered. Different forms of vitamin E exert differential effects on immune cells. Cell-specific effects of vitamin E provide valuable evidence regarding the immunomodulatory mechanisms of vitamin E, but the interplay between immune cells should not be ignored, because interactions between immune cells are critical in the regulation of immune function.

## Figures and Tables

**Table 1:** Modulation of immune responses by vitamin E in animal models.

Species	Dosage and Duration	Form of Vitamin E Used	Results	References
Chicks, female broiler (*n* = 6/group, 6 replicate)	100 mg/kg diet for 21 days	dl-α-tocopheryl acetate	↑Plasma IgM levels at day 21	Dalia et al. 2018 [8]
			↔Splenic expressions of TNF-α, IFN-γ, IL-2, IL-10	
Pregnant cows (*n* = 24/group)	250 IU/day from day 107 of gestation to day 21 of lactation	NA	↑IgG and IgA concentration in sow plasma	Wang et al. 2017 [9]
Domestic cats (39 castrated male and 33 intact female) (*n* = 8/group)	225, 450 mg/kg diet for 28 days	α-tocopherol	↑Lymphocyte proliferation (ConA, PHA)	O’ Brien et al. 2015 [10]
Young and old mice (*n =* 11–13/group)	500 mg/kg diet for 6 weeks	dl-α-tocotrienol	↑Lymphocyte proliferation in old (ConA, PHA)	Ren et al. 2010 [11]
			↑IL-1β production in young	
Young rats (*n =* 6/group)	50, 200 mg/kg diet for 8–10 weeks		↑Lymphocyte proliferation (ConA, LPS)	Bendich et al. 1986 [12]
Old mice (*n =* 10/group)	500 mg/kg diet for 6 weeks	dl-α-tocopheryl acetate	↑Lymphocyte proliferation (ConA, LPS)	Meydani et al. 1986 [13]
			↑DTH response	
			↑IL-2 production	
			↓PGE_2_ production	
Young and old mice (*n =* 5/group)	500 IU (500 mg) for 9 weeks	dl-α-tocopherol acetate	↑Lymphocyte proliferation (ConA) in young	Wakikawa et al. 1999 [14]
			↔Lymphocyte proliferation (ConA) in old	
			↑IFN-γ in young under restraint stress	
Young rats (*n =* 10/group)	50, 100, 250, 500, 2500 mg/kg diet for 7 days	dl-α-tocopheryl acetate	↑Lymphocyte proliferation (>100 mg/kg diet, ConA) (>250 mg/kg diet, LPS)	Moriguchi et al. 1990 [15]
			↑NK activity (>250 mg/kg diet)	
Old rats (*n =* 5/group)	585 mg/kg diet for 12 months	dl-α-tocopheryl nicotinate	↑Lymphocyte proliferation (ConA, PHA)	Sakai S & Moriguchi 1997 [16]
			↑IL-2 production	
Young calves (*n =* 8/group)	125, 250, 500 IU (125, 250, 500 mg)/day for 24 weeks	dl-α-tocopheryl acetate	↑Lymphocyte proliferation (PHA, ConA, pokeweed mitogen)	Reddy et al. 1987 [17]
			↑Antibovine herpesvirus Ab titer to booster in 125 IU/day group	
Young mice (*n =* 8/group)	200 mg/kg diet for 6–12 weeks	α-tocopheryl acetate	↑Ab response	Tanake et al. 1979 [18]
			↑Helper T cell activity	
Mice (*n =* 10/group)	500 mg/kg diet for 6 months	α-tocopherol acetate (Tekland, Madison, WI)	↓IL-6 and PGEs (unstimulated) production by macrophages	Beharka et al. 2000 [19]
			↓Nitric oxide production (LPS) by macrophages	

Ab, antibody; ConA, concanavalin A; IFN-γ, interferon-γ; LPS, lipopolysaccharide; PGE_2_, prostaglandin E_2_; PHA, phytohemagglutinin; TNF, Tumor necrosis factor.

**Table 2:** Modulation of immune responses by vitamin E in humans.

Subjects	Age	Amount and Duration of Supplementation	Form of Vitamin E Used	Effects on Immune Function	References
Young (*n =* 5) and senior athletes (*n =* 5)	18–25, 35–57	4.6 ± 0.3 mg/100 mL of vitamin E-enriched beverage 5 days/week for 5 weeks	α-tocopherol acetate	↑15LOX2, TNF-α expression	Capo et al. 2016 [20]
Healthy women (*n =* 108)	18–25	400 mg TRF/day for 56 days	d-α-tocotrienol	↑IL-4 (TT vaccine), IFN-γ (ConA)	Mahalingam et al. 2011 [21]
			d-γ-tocotrienol		
			d-δ-tocotrienol	↓IL-6 (LPS)	
			d-α-tocopherol		
Healthy men and women (*n =* 19, 34)	20–50	200 mg/day for 56 days	α-tocopherol	↔IL-4, IFN-γ production (ConA)	Radhakrishnan et al. 2009 [22]
Adult males and young boys (*n =* 18)	25–30, 13–18	300 mg/day for 3 weeks	dl-α-tocopheryl acetate	↓Lymphocyte proliferation (PHA)	Prasad 1980 [23]
				↔DTH	
				↓Bactericidal activity	
Institutionalized adult males and females (*n =* 103)	24–104	200, 400 mg/day for 6 months	α-tocopherol acetate	↔Ab development to influenza virus	Harman and Miller 1986 [24]
Healthy elderly males and females (*n =* 32)	≥60	800 mg/day for 30 days	dl-α-tocopheryl acetate	↑Lymphocyte proliferation (ConA)	Meydani et al. 1990 [25]
				↑DTH	
				↑IL-2 production (ConA)	
				↓PGE_2_ production (PHA)	
Eldery males and females (*n =* 74)	≥65	100 mg/day for 3 months	dl-α-tocopheryl acetate	↔Lymphocyte proliferation (ConA, PHA)	De Waart et al. 1997 [26]
				↔IgG, IgA levels	
Healthy elderly males and females (*n =* 88)	≥65	60, 200, 800 mg/day for 235 days	dl-α-tocopherol	↑DTH and antibody titer to hepatitis B with 200, 800 mg	Meydani et al. 1997 [27]
Healthy elderly males and females (*n =* 161)	65–80	50, 100 mg/day for 6 months	dl-α-tocopheryl acetate	↑No. of positive DTH reaction with 100 mg	Pallast et al. 1999 [28]
				↑dDiameter of induration of DTH reaction in a subgroup supplemented with 100 mg	
				↔IL-2 production	
				↓IFN-γ production	
Healthy young adults (*n =* 31) and premature infants (*n =* 10)	24–31	600 mg/day for 3 months40 mg/kg body weight for 8–14 days		↓Chemiluminescence	Okano et al. 1990 [29]
Cigarette smoker (*n =* 60)	33 ± 4	900 IU/day for 6 weeks		↓Chemiluminescence	Richards et al. 1990 [30]
Healthy males (*n =* 40)	24–57	200 mg/day for 4 months	*all-rac*-α-tocopherol	Prevented fish-oil-induced suppression of ConA mitogenesis	Kramer et al. 1991 [31]
Healthy elderly (*n =* 40)	>65	100, 200, or 400 mg/day for 3 months	dl-α-tocopherol	↑DTH (maximal diameter) in 100, 200, 400 mg groups	Wu et al. 2006 [32]
				↑Lymphocyte proliferation (ConA) in 200 mg group	
Sedentary young and elderly males (*n =* 21)	22–29, 55–74	800 IU (727 mg)/day for 48 days	dl-α-tocopherol	↓IL-6 secretion	Cannon et al. 1991 [33]
				↓Exercise-enhanced IL-1β secretion	

ConA, concanavalin A; DTH, delayed type hypersensitivity; IFN-γ, interferon-γ; 15LOX2, 15-lipoxygenase-2; PGE_2_, prostaglandin E_2_; PHA, phytohemagglutinin; TRF, tocotrienol-rich fraction; TT vaccine, tetanous toxoid vaccine.

**Table 3:** Effects of vitamin E supplementation on infectious diseases in animal models.

Subjects	Age	Dose and Duration of Supplementation	Form of Vitamin E Used	Infection Organism and Route of Infection	Results: Effects of Vitamin E Supplementation	References
Mice BALB/c (*n =* 3–6/group)	6 months	100 mg/kg for 8 days before MRSA-challenge	δ-, γ-Tocotrienol	MRSA, inoculated onto superficial surgical wounds	Higher NK cytotoxicity	Pierpaoli et al. 2017 [34]
					Higher IL-24 mRNA expression levels	
Young and aged male mice C57BL/6 (*n =* 6/group)	2, 22–26 months	500 mg/kg for 4 weeks prior to infection	d-α-tocopheryl acetate	*Streptococcus pneumoniae*, intra-tracheally injected	1000-fold fewer bacteria in their lung	Bou Ghanem et al. 2015 [35]
					Age-associated higher production of proinflammatory cytokines (TNF-, IL-6) were reduced	
					3-fold reduction in the number of PMNs	
Worm-free lambs (*n* =10/group)	28–32 weeks	5.3 IU (3.56 mg)/kg BW for 12 weeks	d-α-tocopherol	*H. contortus* L3 larvae, route NA	No difference in serum IgG or peripheral mRNA expression of IL-4 or IFN-γ	De Wolf et al. 2014 [36]
					Lower PCV, FEC, and worm burden	
Male mice BALB/c (*n =* 6–7/group)	At weaning	Deficient, Adequate (38.4 mg/kg diet), or Supplemented (384 mg/kg diet) for 4 weeks	dl-α-tocopheryl acetate	HSV-1, intranasally	Higher viral titre and ILβ, TNF-α, RANTES in the brain with E deficiency	Sheridan & Beck. 2008 [37]
					No difference in expressions of IL-6, TNFα, IL-1β, and IL-10 between adequate and supplemented	
Mice C57BL (*n =* 6–9/group)	22 months	500 mg/kg diet for 8 weeks	dl-α-tocopherol acetate	Influenza by nasal inoculation	Lower viral titer	Han et al. 2000 [6]
					Higher IL-2 and IFN-γ production	
Mice, C57BL/6 (*n =* 4–9)	22 months	500mg/kg diet for 6 weeks	dl-α-tocopherol acetate	Influenza A/PC/1/73 (H3N2) by nasal inoculation	Lower viral titre	Hayek et al. 1997 [7]
Mice, C57BL/6 (*n =* 6)	5 weeks	160 IU/L liquid diet for 4, 8, 12, 16 weeks	*all-rac*-α-tocopheryl acetate	Murine LP-BM5 leukaemia retrovirus by IP injection	Restored IL-2 and IFN-γ production by splenocytes following infection	Wang et al. 1994 [38]
Calves, Holstein (*n =* 7)	1d	1400 or 2800 mg orally once per week, 1400 mg injection once per week for 12 weeks	dl-α-tocopheryl acetate	Bovine rhinotracheitis virus, in vitro	Serum from vitamin E-supplemented calves inhibited the replication of bovine rhinotracheitis virus in vitro	Reddy et al. 1986 [39]
Mice, Swiss Webster (*n =* 10)	4 weeks	180 mg/kg diet for 4 weeks	dl-α-tocopheryl acetate	*Diplococcus pneumoniae* type I by IP injection	Higher survival	Heinzerling et al. 1974a [5]
Mice, BALB/C (*n =* 25)	NA	25 or 250 mg/kg bw orally for 4 days, starting 2 days before burn injury	dl-α-tocopheryl acetate	*Pseudomonas aeruginosa,* subeschar injection to burned mice	Lower mortality rate	Fang et al. 1990 [40]
Mice, BALB/C (NA)	3 weeks	4000mg/kg diet for 2, 4, or 14 weeks	Vitamin E injectable (aqueous)	*Listeria monocytogenes* by IP injection	No difference in resistance	Watson & Petro 1982 [41]
Rats, Sprague-Dawley (*n =* 6)	3 weeks	180 mg/kg diet + 6000 IU vitamin A/kg diet for 6 weeks	dl-α-tocopheryl acetate	*Mycoplasma pulmonis* by aerosol	Higher resistance to infection	Tvedten et al. 1973 [42]
Lambs (*n =* 10)	NA	1000 IU orally, 300 mg/kg diet for 23 days	dl-α-tocopheryl acetate	Chlamydia by intratracheal inoculation	Faster recovery (higher food intake and weight gains)	Stephens et al. 1979 [43]
Turkey, broadbreasted white poults (*n =* 6)	1 day	500 mg/kg diet for 14 days before infection and 18–21 days after infection	dl-α-tocopheryl acetate	*Histomonas meleagridis*, oral	No effect on mortality by vitamin E supplementation alone	Schildknecht & Squibb 1979 [44]
					Lower mortality and lesion score in combination with ipronidazole	
Pigs (*n =* 6)	NA	200 mg/pig per day for 59 days before infection and 22 days after infection	dl-α-tocopheryl acetate	*Treponema hyodysenteriae*, oral	Improved weight gain and recovery rate	Teige et al. 1982 [45]
					No beneficial effect on appetite and diarrhoea	
Sheep (*n =* 12)	3–6 months	300 mg/kg diet starting 2 weeks before first vaccination	dl-α-tocopheryl acetate	*Clostridium perfringens* type D by IV injection after two IM vaccinations	Higher Ab titre	Tengerdy et al. 1983 [46]
					Fail to prove beneficial effect of vitamin E on protection (none of the vaccinated lambs died)	
Cows (*n =* 20)	NA	740 mg/cow per day, duration NA	dl-α-tocopheryl acetate	Natural occurrence of clinical mastitis due to *Streptococci, Coliform, Staphylococci, Clostridium bovis*	Lower clinical cases of mastitis	Smith et al. 1984 [47]
Chicks, broiler (*n =* 12–14)	1day	150 mg or 300mg/kg diet for 2 weeks before infection	dl-α-tocopheryl acetate	*Escherichia coli*, orally and post-thoracic air sac	Lower mortality	Heinzerling et al. 1974b [48]
					Higher Ab titre	
Chicks, broiler (*n =* 10)	1 day	300 mg/kg diet for 6 weeks, starting 3 weeks before first infection	dl-α-tocopheryl acetate	*E. coli*, post-thoracic air sac	Lower mortality	Tengerdy & Nockels 1975 [49]
Chicks, Leghorn (*n =* 22)	1 day	300 mg/kg diet for 4 weeks before infection	dl-α-tocopheryl acetate	*E. coli* by IV injection	Lower mortality	Likoff et al. 1981 [50]
Pigs (*n =* 10)	6–8 weeks	100, 000 mg/t diet for 10 weeks, starting 2 weeks before infection	Vitamin E; Tompson-Hayward, Minneapolis, MN, USA	*E. coli* by IM injection	Higher serum Ab titre	Ellis & Vorhies 1976 [51]

Ab, antibody; FEC, fecal egg count; HSV, Herpes simplex virus; MRSA, IFN-γ, interferon-γ; IM, intramuscular; IV, intravenous; Methicillin-resistant *Staphylococcus aureus*; NK, natural killer; PCV, packed cell volume; PMN, polymorphonuclear leukocyte, RANTES, regulated on activation, normal T cell expressed and secreted; TNF-α, tumor necrosis factor-α.

**Table 4:** Effects of vitamin E supplementation on infectious diseases in humans.

Subjects	Age	Dose and Duration of Supplementation	Form of Vitamin E Used	Infection Organism and Route of Infection	Results: Effects of Vitamin E Supplementation	References
Male smoker	50–69	50 mg/d for median of 6 years	dl-α-tocopheryl acetate	Natural incidence of pneumonia	69% Lower incidence of pneumonia among subgroups including participants who smoked 5–19 cigarettes per day at baseline and exercised at leisure time	Hemila et al. 2016 [52]
					14% Lower incidence of pneumonia among subgroups including participants who smoked ≥20 cigarettes per day at baseline and did not exercise	
HIV-infected pregnant Tanzanian women	25.4	30 mg during pregnancy (multivitamin form with 20 mg vitamins B1, 20 mg B2, 25 mg B6, 100 mg niacin, 50 μg B12, 500 mg C, and 800 μg folic acid)	NA	Natural incidence of malaria after having received malaria prophylaxis during pregnancy	Lower incidence of presumptive clinical malaria, but higher risk of any malaria parasitemia	Olofin et al. 2014 [53]
Patients with HCV-related cirrhosis	54–75	900 IU (604.03 mg for d- or 818.18 mg for dl-)/day for 6 months	α-tocopherol	Natural incidence of cirrhosis	Reduced glutathione (GSH) and glutathione peroxidase, which are significantly lower in cirrhotic patients (*p* < 0.05), were comparably improved by vitamin E regimens	Marotta et al. 2007 [54]
Patients with chronic HCV	18–75	945 IU (634.23 mg)/day for 6 months with 500 mg ascorbic acid and 200 μg of selenium	d-α-tocopherol	Natural incidence of HCV	No difference in median log plasma HCV-RNA	Groenbak et al. 2006 [55]
Nursing home residents	>65	200 IU/day for 1 year	dl-α-tocopherol	Natural incidence of respiratory infections	Fewer numbers of subjects with all and upper respiratory infections	Meydani et al. 2004 [56]
					Lower incidence of common cold	
					No effect on lower respiratory infection	
Male smokers	50–69	50 mg/day during 4-year follow-up	α-tocopherol	Natural incidence of common cold episodes	Lower incidence of common cold	Hemila et al. 2002 [57]
					Reduction was greatest among older city dwellers who smoked fewer than 15 cigarettes per day	
Male smokers	50–69 years	50 mg/day for median of 6.1 years	dl-α-tocopheryl acetate	Natural incidence of pneumonia	No overall effect on the incidence of pneumonia.	Hemila et al. 2004 [58]
					Lower incidence of pneumonia among the subjects who had initiated smoking at a later age (>21)	
Non-institutionalized individuals	>60 years	200 mg/day for median of 441 days	α-tocopherol acetate	Natural incidence and severity of self-reported acute respiratory tract infections	No effect on incidence and severity of acute respiratory tract infections	Graat et al. 2002 [59]

HCV, hepatitis C virus.

## References

[1] Traber M.G. (2007). Vitamin E regulatory mechanisms. Annu. Rev. Nutr..

[2] Sheppard A.J., Pennington J.A.T., Weihrauch J.L., Packer L., Fuchs J. (1980). Analysis and distribution of vitamin E in vegetable oils and foods. Vitamin E in Health and Disease.

[3] Traber M.G., Atkinson J. (2007). Vitamin E, antioxidant and nothing m more. Free Rad. Biol. Med..

[4] Zingg J.M. (2015). Vitamin E: A role in signal transduction. Annu. Rev. Nutr..

[5] Heinzerling R.H., Tengerdy R.P., Wick L.L., Lueker D.C. (1974). Vitamin E protects mice against Diplococcus pneumoniae type I infection. Infect. Immun..

[6] Han S.N., Wu D., Ha W.K., Beharka A., Smith D.E., Bender B.S., Meydani S.N. (2000). Vitamin E supplementation increases T helper 1 cytokine production in old mice infected with influenza virus. Immunology.

[7] Hayek M.G., Taylor S.F., Bender B.S., Han S.N., Meydani M., Smith D.E., Eghtesada S., Meydani S.N. (1997). Vitamin E supplementation decreases lung virus titers in mice infected with influenza. J. Infect. Dis..

[8] Dalia A.M., Loh T.C., Sazili A.Q., Jahromi M.F., Samsudin A.A. (2018). Effects of vitamin E, inorganic selenium, bacterial organic selenium, and their combinations on immunity response in broiler chickens. BMC Vet. Res..

[9] Wang L., Xu X., Su G., Shi B., Shan A. (2017). High concentration of vitamin E supplementation in sow diet during the last week of gestation and lactation affects the immunological variables and antioxidative parameters in piglets. J. Dairy Res..

[10] O’Brien T., Thomas D.G., Morel P.C., Rutherfurd-Markwick K.J. (2015). Moderate dietary supplementation with vitamin E enhances lymphocyte functionality in the adult cat. Res. Vet. Sci..

[11] Ren Z., Pae M., Dao M.C., Smith D., Meydani S.N., Wu D. (2010). Dietary supplementation with tocotrienols enhances immune function in C57BL/6 mice. J. Nutr..

[12] Bendich A., Gabriel E., Machlin L.J. (1986). Dietary vitamin E requirement for optimum immune responses in the rat. J. Nutr..

[13] Meydani S.N., Meydani M., Verdon C.P., Shapiro A.A., Blumberg J.B., Hayes K.C. (1986). Vitamin E supplementation suppresses prostaglandin E1(2) synthesis and enhances the immune response of aged mice. Mech. Ageing Dev..

[14] Wakikawa A., Utsuyama M., Wakabayashi A., Kitagawa M., Hirokawa K. (1999). Vitamin E enhances the immune functions of young but not old mice under restraint stress. Exp. Gerontol..

[15] Moriguchi S., Kobayashi N., Kishino Y. (1990). High dietary intakes of vitamin E and cellular immune functions in rats. J. Nutr..

[16] Sakai S., Moriguchi S. (1997). Long-term feeding of high vitamin E diet improves the decreased mitogen response of rat splenic lymphocytes with aging. J. Nutr. Sci. Vitaminol..

[17] Reddy P.G., Morrill J.L., Minocha H.C., Stevenson J.S. (1987). Vitamin E is Immunostimulatory in calves. J. Dairy Sci..

[18] Tanaka J., Fujiwara H., Torisu M. (1979). Vitamin E and immune response. I. Enhancement of helper T cell activity by dietary supplementation of vitamin E in mice. Immunology.

[19] Beharka A.A., Han S.N., Adolfsson O., Wu D., Lipman R., Smith D., Cao G., Meydani M., Meydani S.N. (2000). Long-term dietary antioxidant supplementation reduces production of selected inflammatory mediators by murine macrophages. Nutr. Res..

[20] Capó X., Martorell M., Sureda A., Riera J., Drobnic F., Tur J.A., Pons A. (2016). Effects of Almond- and Olive Oil-Based Docosahexaenoic- and Vitamin E-Enriched Beverage Dietary Supplementation on Inflammation Associated to Exercise and Age. Nutrients..

[21] Mahalingam D., Radhakrishnan A.K., Amom Z., Ibrahim N., Nesaretnam K. (2011). Effects of supplementation with tocotrienol-rich fraction on immune response to tetanus toxoid immunization in normal healthy volunteers. Eur. J. Clin. Nutr..

[22] Radhakrishnan A.K., Lee A.L., Wong P.F., Kaur J., Aung H., Nesaretnam K. (2009). Daily supplementation of tocotrienol-rich fraction or alpha-tocopherol did not induce immunomodulatory changes in healthy human volunteers. Br. J. Nutr..

[23] Prasad J.S. (1980). Effect of vitamin E supplementation on leukocyte function. Am. J. Clin. Nutr..

[24] Harman D., Miller R.W. (1986). Effect of vitamin E on the immune response to influenza virus vaccine and the incidence of infectious disease in man. Age.

[25] Meydani S.N., Barklund M.P., Liu S., Meydani M., Miller R.A., Cannon J.G., Morrow F.D., Rocklin R., Blumberg J.B. (1990). Vitamin E supplementation enhances cell-mediated immunity in healthy elderly subjects. Am. J. Clin. Nutr..

[26] De Waart F.G., Portengen L., Doekes G., Verwaal C.J., Kok F.J. (1997). Effect of 3 months vitamin E supplementation on indices of the cellular and humoral immune response in elderly subjects. Br. J. Nutr..

[27] Meydani S.N., Meydani M., Blumberg J.B., Leka L.S., Siber G., Loszewski R., Thompson C., Pedrosa M.C., Diamond R.D., Stollar B.D. (1997). Vitamin E supplementation and in vivo immune response in healthy elderly subjects. A randomized controlled trial. JAMA.

[28] Pallast E.G., Schouten E.G., de Waart F.G., Fonk H.C., Doekes G., von Blomberg B.M., Kok F.J. (1999). Effect of 50- and 100-mg vitamin E supplements on cellular immune function in noninstitutionalized elderly persons. Am. J. Clin. Nutr..

[29] Okano T., Tamai H., Mino M. (1991). Superoxide generation in leukocytes and vitamin E. Int. J. Vitam. Nutr. Res..

[30] Richards G.A., Theron A.J., van Rensburg C.E., van Rensburg A.J., van der Merwe C.A., Kuyl J.M., Anderson R. (1990). Investigation of the effects of oral administration of vitamin E and beta-carotene on the chemiluminescence responses and the frequency of sister chromatid exchanges in circulating leukocytes from cigarette smokers. Am. Rev. Respir. Dis..

[31] Kramer T.R., Schoene N., Douglass L.W., Judd J.T., Ballard-Barbash R., Taylor P.R., Bhagavan H.N., Nair P.P. (1991). Increased vitamin E intake restores fish-oil-induced suppressed blastogenesis of mitogen-stimulated T lymphocytes. Am. J. Clin. Nutr..

[32] Wu D., Han S.N., Meydani M., Meydani S.N. (2006). Effect of concomitant consumption of fish oil and vitamin E on T cell mediated function in the elderly: A randomized double-blind trial. J. Am. Coll. Nutr..

[33] Cannon J.G., Meydani S.N., Fielding R.A., Fiatarone M.A., Meydani M., Farhangmehr M., Orencole S.F., Blumberg J.B., Evans W.J. (1991). Acute phase response in exercise. II. Associations between vitamin E, cytokines, and muscle proteolysis. Am. J. Physiol..

[34] Pierpaoli E., Orlando F., Cirioni O., Simonetti O., Giacometti A., Provinciali M. (2017). Supplementation with tocotrienols from Bixa orellana improves the in vivo efficacy of daptomycin against methicillin-resistant Staphylococcus aureus in a mouse model of infected wound. Phytomedicine.

[35] Bou Ghanem E.N., Clark S., Du X., Wu D., Camilli A., Leong J.M., Meydani S.N. (2015). The α-tocopherol form of vitamin E reverses age-associated susceptibility to streptococcus pneumoniae lung infection by modulating pulmonary neutrophil recruitment. J. Immunol..

[36] De Wolf B.M., Zajac A.M., Hoffer K.A., Sartini B.L., Bowdridge S., LaRoith T., Petersson K.H. (2014). The effect of vitamin E supplementation on an experimental Haemonchus contortus infection in lambs. Vet. Parasitol..

[37] Sheridan P.A., Beck M.A. (2008). The immune response to herpes simplex virus encephalitis in mice is modulated by dietary vitamin E. J. Nutr..

[38] Wang Y., Huang D.S., Eskelson C.D., Watson R.R. (1994). Long-Term Dietary Vitamin E Retards Development of Retrovirus-Induced Disregulation in Cytokine Production. Clin. Immunol. Immunopathol..

[39] Reddy P.G., Morrill J.L., Minocha H.C., Morrill M.B., Dayton A.D., Frey R.A. (1986). Effect of supplemental vitamin E on the immune system of calves. J. Dairy Sci..

[40] Fang C.H., Peck M.D., Alexander J.W., Babcock G.F., Warden G.D. (1990). The effect of free radical scavengers on outcome after infection in burned mice. J. Trauma..

[41] Watson R., Petro T.M. (1982). Cellular immune response, corticosteroid levels and resistance to Listeria monocytogenes and murine leukemia in mice fed a high vitamin E diet. N.Y. Acad. Sci..

[42] Tvedten H.W., Whitehair C.K., Langham R.F. (1973). Influence of vitamins A and E on gnotobiotic and conventionally maintained rats exposed to Mycoplasma pulmonis. J. Am. Vet. Med. Assoc..

[43] Stephens L.C., McChesney A.E., Nockels C.F. (1979). Improved recovery of vitamin E-treated lambs that have been experimentally infected with intratracheal Chlamydia. Br. Vet. J..

[44] Schildknecht E.G., Squibb R.L. (1979). The effect of vitamins A, E and K on experimentally induced histomoniasis in turkeys. Parasitology.

[45] Teige J., Tollersrud S., Lund A., Larsen H.J. (1982). Swine dysentery: The influence of dietary vitamin E and selenium on the clinical and pathological effects of Treponema hyodysenteriae infection in pigs. Res. Vet. Sci..

[46] Tengerdy R.P., Meyer D.L., Lauerman L.H., Lueker D.C., Nockels C.F. (1983). Vitamin E-enhanced humoral antibody response to Clostridium perfringens type D in sheep. Br. Vet. J..

[47] Smith K.L., Harrison J.H., Hancock D.D., Todhunter D.A., Conrad H.R. (1984). Effect of vitamin E and selenium supplementation on incidence of clinical mastitis and duration of clinical symptoms. J. Dairy Sci..

[48] Heinzerling R.H., Nockels C.F., Quarles C.L., Tengerdy R.P. (1974). Protection of chicks against *E.coli* infection by dietary supplementation with vitamin E. Proc. Soc. Exp. Biol. Med..

[49] Tengerdy R.P., Nockels C.F. (1975). Vitamin E or vitamin A protects chickens against E. coli infection. Poult. Sci..

[50] Likoff R.O., Guptill D.R., Lawrence L.M., McKay C.C., Mathias M.M., Nockels C.F., Tengerdy R.P. (1981). Vitamin E and aspirin depress prostaglandins in protection of chickens against Escherichia coli infection. Am. J. Clin. Nutr..

[51] Ellis R.P., Vorhies M.W. (1976). Effect of supplemental dietary vitamin E on the serologic response of swine to an Escherichia coli bacterin. J. Am. Vet. Med. Assoc..

[52] Hemilä H. (2016). Vitamin E administration may decrease the incidence of pneumonia in elderly males. Clin. Interv. Aging.

[53] Olofin I.O., Spiegelman D., Aboud S., Duggan C., Danaei G., Fawzi W.W. (2014). Supplementation with multivitamins and vitamin A and incidence of malaria among HIV-infected Tanzanian women. J. Acquir. Immune Defic. Syndr..

[54] Marotta F., Yoshida C., Barreto R., Naito Y., Packer L. (2007). Oxidative-inflammatory damage in cirrhosis: Effect of vitamin E and a fermented papaya preparation. J. Gastroenterol. Hepatol..

[55] Groenbaek K., Friis H., Hansen M., Ring-Larsen H., Krarup H.B. (2006). The effect of antioxidant supplementation on hepatitis C viral load, transaminases and oxidative status: A randomized trial among chronic hepatitis C virus-infected patients. Eur. J. Gastroenterol. Hepatol..

[56] Meydani S.N., Leka L.S., Fine B.C., Dallal G.E., Keusch G.T., Singh M.F., Hamer D.H. (2004). Vitamin E and respiratory tract infections in elderly nursing home residents: A randomized controlled trial. JAMA.

[57] Hemilä H., Kaprio J., Albanes D., Heinonen O.P., Virtamo J. (2002). Vitamin C, vitamin E, and beta-carotene in relation to common cold incidence in male smokers. Epidemiology.

[58] Hemilä H., Virtamo J., Albanes D., Kaprio J. (2004). Vitamin E and beta-carotene supplementation and hospital-treated pneumonia incidence in male smokers. Chest.

[59] Graat J.M., Schouten E.G., Kok F.J. (2002). Effect of daily vitamin E and multivitamin-mineral supplementation on acute respiratory tract infections in elderly persons: A randomized controlled trial. JAMA.

[60] Mosser D.M., Edwards J.P. (2008). Exploring the full spectrum of macrophage activation. Nat. Rev. Immunol..

[61] Meydani S.N., Han S.N., Wu D. (2005). Vitamin E and immune response in the aged: Molecular mechanisms and clinical implications. Immunol. Rev..

[62] Beharka A.A., We D., Han S.N., Meydani S.N. (1997). Macrophage prostaglandin production contributes to the age-associated decrease in T cell function which is reversed by the dietary antioxidant vitamin E. Mech. Age. Dev..

[63] Hayek M.G., Mura C., Wu D., Beharka A.A., Han S.N., Paulson K.E., Hwang D., Meydani S.N. (1997). Enhanced expression of inducible cyclooxygenase with age in murine macrophages. J. Immunol..

[64] Wu D., Mura C., Beharka A.A., Han S.N., Paulson K.E., Hwang D., Meydani S.N. (1998). Age-associated increase in PGE2 synthesis and COX activity in murine macrophages is reversed by vitamin E. Am. J. Physiol..

[65] Beharka A.A., Wu D., Serafini M., Meydani S.N. (2002). Mechanism of vitamin E inhibition of cyclooxygenase activity in macrophages from old mice: Role of peroxynitrite. Free. Radic. Biol. Med..

[66] Sorokin A. (2016). Nitric Oxide Synthase and Cyclooxygenase Pathways: A Complex Interplay in Cellular Signaling. Curr. Med. Chem..

[67] Dworski R., Han W., Blackwell T.S., Hoskins A., Freeman M.L. (2011). Vitamin E prevents NRF2 suppression by allergens in asthmatic alveolar macrophages in vivo. Free Radic. Biol. Med..

[68] Adachi N., Migita M., Ohta T., Higashi A., Matsuda I. (1997). Depressed natural killer cell activity due to decreased natural killer cell population in a vitamin E-deficient patient with Shwachman syndrome: Reversible natural killer cell abnormality by alpha-tocopherol supplementation. Eur. J. Pediatr..

[69] Ravaglia G., Forti P., Maioli F., Bastagli L., Facchini A., Mariani E., Savarino L., Sassi S., Cucinotta D., Lenaz G. (2000). Effect of micronutrient status on natural killer cell immune function in healthy free-living subjects aged ≥90 y. Am. J. Clin. Nutr..

[70] Hanson M.G., Ozenci V., Carlsten M.C., Glimelius B.L., Frödin J.E., Masucci G., Malmberg K.J., Kiessling R.V. (2007). A short-term dietary supplementation with high doses of vitamin E increases NK cell cytolytic activity in advanced colorectal cancer patients. Cancer Immunol. Immunother..

[71] Stiff A., Trikha P., Mundy-Bosse B., McMichael E., Mace T.A., Benner B., Kendra K., Campbell A., Gautam S., Abood D. (2018). Nitric Oxide Production by Myeloid-Derived Suppressor Cells Plays a Role in Impairing Fc Receptor-Mediated Natural Killer Cell Function. Clin. Cancer Res..

[72] Ganguly D., Haak S., Sisirak V., Reizis B. (2013). The role of dendritic cells in autoimmunity. Nat. Rev. Immunol..

[73] Pearce E.J., Everts B. (2015). Dendritic cell metabolism. Nat. Rev. Immunol..

[74] Alloatti A., Kotsias F., Magalhaes J.G., Amigorena S. (2016). Dendritic cell maturation and cross-presentation: Timing matters!. Immunol. Rev..

[75] Tan P.H., Sagoo P., Chan C., Yates J.B., Campbell J., Beutelspacher S.C., Foxwell B.M., Lombardi G., George A.J. (2005). Inhibition of NF-kappa B and oxidative pathways in human dendritic cells by antioxidative vitamins generates regulatory T cells. J. Immunol..

[76] Xuan N.T., Trang P.T., Van Phong N., Toan N.L., Trung D.M., Bac N.D., Nguyen V.L., Hoang N.H., van Hai N. (2016). Klotho sensitive regulation of dendritic cell functions by vitamin E. Biol. Res..

[77] Buendía P., Ramírez R., Aljama P., Carracedo J. (2016). Klotho Prevents Translocation of NFκB. Vitam. Horm..

[78] Abdala-Valencia H., Berdnikovs S., Soveg F.W., Cook-Mills J.M. (2014). α-Tocopherol supplementation of allergic female mice inhibits development of CD11c+CD11b+ dendritic cells in utero and allergic inflammation in neonates. Am. J. Physiol. Lung Cell. Mol. Physiol..

[79] Abdala-Valencia H., Soveg F., Cook-Mills J.M. (2016). γ-Tocopherol supplementation of allergic female mice augments development of CD11c+CD11b+ dendritic cells in utero and allergic inflammation in neonates. Am. J. Physiol. Lung Cell. Mol. Physiol..

[80] Molano A., Meydani S.N. (2012). Vitamin E, signalosomes and gene expression in T cells. Mol. Aspects. Med..

[81] Adolfsson O., Huber B.T., Meydani S.N. (2001). Vitamin E-enhanced IL-2 production in old mice: Naive but not memory T cells show increased cell division cycling and IL-2-producing capacity. J. Immunol..

[82] Marko M.G., Ahmed T., Bunnell S.C., Wu D., Chung H., Huber B.T., Meydani S.N. (2007). Age-associated decline in effective immune synapse formation of CD4(+) T cells is reversed by vitamin E supplementation. J. Immunol..

[83] Marko M.G., Pang H.J., Ren Z., Azzi A., Huber B.T., Bunnell S.C., Meydani S.N. (2009). Vitamin E reverses impaired linker for activation of T cells activation in T cells from aged C57BL/6 mice. J. Nutr..

[84] Han S.N., Adolfsson O., Lee C.K., Prolla T.A., Ordovas J., Meydani S.N. (2006). Age and vitamin E-induced changes in gene expression profiles of T cells. J. Immunol..

[85] Pelizon C. (2003). Down to the origin: Cdc6 protein and the competence to replicate. Trends Cell Biol..

[86] Li-Weber M., Giaisi M., Treiber M.K., Krammer P.H. (2002). Vitamin E inhibits IL-4 gene expression in peripheral blood T cells. Eur. J. Immunol..

[87] Malmberg K.J., Lenkei R., Petersson M., Ohlum T., Ichihara F., Glimelius B., Frödin J.E., Masucci G. (2002). A short-term dietary supplementation of high doses of vitamin E increases T helper 1 cytokine production in patients with advanced colorectal cancer. Clin. Cancer Res..

[88] Li-Weber M., Weigand M.A., Giaisi M., Süss D., Treiber M.K., Baumann S., Ritsou E., Breitkreutz R., Krammer P.H. (2002). Vitamin E inhibits CD95 ligand expression and protects T cells from activation-induced cell death. J. Clin. Invest..

[89] Neuzil J., Svensson I., Weber T., Weber C., Brunk U.T. (1999). α-tocopheryl succinate-induced apoptosis in Jurkat T cells involves caspase-3 activation, and both lysosomal and mitochondrial destabilisation. FEBS Lett..

